# Editorial: Clinical impact of fast platforms and laboratory automation for the rapid diagnosis of infectious diseases and detection of antimicrobial resistance determinants

**DOI:** 10.3389/fcimb.2023.1321663

**Published:** 2023-12-04

**Authors:** Fabio Arena, Paola Bernaschi, Antonella Mencacci

**Affiliations:** ^1^ Department of Clinical and Experimental Medicine, University of Foggia, Foggia, Italy; ^2^ Microbiology and Diagnostic Immunology Unit, Bambino Gesù Children’s Hospital, Istituto di Ricovero e Cura a Carattere Scientifico (IRCCS), Rome, Italy; ^3^ Microbiology and Clinical Microbiology, Department of Medicine and Surgery, University of Perugia, Perugia, Italy; ^4^ Microbiology, Perugia General Hospital, Perugia, Italy

**Keywords:** clinical outcome, innovation, next-generation sequencing, artificial intelligence, new diagnostics

Infectious diseases remain a significant global health threat, exacerbated by the emergence of antimicrobial resistance (Centers for Disease Control and Prevention, 2019; Baquero et al., 2021; European Centre for Disease Prevention and Control, 2022; World Health Organization, 2022). Accurate and timely microbiological diagnosis is vital for effective patient management, infection control, and antimicrobial stewardship (Morency-Potvin et al., 2019).

The availability of fast platforms and laboratory automation have revolutionized clinical microbiology improving the speed and precision of diagnosing infectious diseases (Özenci and Rossolini, 2019; Trotter et al., 2019). Traditional diagnostic methods, such as culture-based techniques, are time-consuming and may take several days to yield results. In contrast, automated platforms like polymerase chain reaction (PCR) and next-generation sequencing (NGS) can rapidly identify pathogens directly from clinical specimens. This rapidity allows for the prompt initiation of appropriate treatment, reducing the risk of complications and transmission to other patients, all while aligning with the principles of diagnostic stewardship (Dien Bard and McElvania, 2020; Hilt and Ferrieri, 2022).

Diagnostic stewardship is a healthcare concept and practice that focuses on the responsible and judicious use of diagnostic tests and procedures to improve patient care, enhance clinical outcomes, and optimize resource utilization. It is closely related to antimicrobial stewardship, which primarily deals with the appropriate use of antibiotics to combat antimicrobial resistance. Diagnostic stewardship, on the other hand, encompasses a broader spectrum of diagnostic tests and procedures beyond just antibiotics (Patel and Fang, 2018).

Prioritizing laboratory practices in a patient-oriented approach can be used to optimize technology advances for improved patient care. Laboratory automation optimizes workflow in clinical microbiology laboratories, reducing the manual labor needed for sample processing and analysis. This not only expedites diagnostic turnaround times but also maximizes resource utilization. Automation diminishes the risk of human error, enhances result reproducibility, and frees laboratory personnel to concentrate on more intricate tasks. All these aspects are discussed in a “Perspective” paper published in this Research Topic (Menacci et al.).

Beside this, several contributions in this Research Topic have focused on how innovative and rapid diagnostic approaches could improve the diagnosis of neglected infective diseases or the detection of slow growing/fastidious pathogens, enhancing the quality of care provided to patients.

For example, an “Original research” paper described the functioning and the performances of a new DNA microarray chip assay which provides a simple, rapid, high-throughput, and reliable method for the diagnosis of cutaneous mycobacterial infections with potential for clinical application (Yu et al.). In another research paper, a recombinase-aided amplification assay targeting the 16S rRNA gene was developed for rapid detection of *Burkholderia cepacia* complex in patients with an uncharacterized infection who are immunocompromised or have underlying diseases, thereby providing guidance for effective treatment (Fu et al.). Finally, a novel automated antifungal susceptibility testing system, Droplet 48, which detects the fluorescence of microdilution wells in real time and fits growth characteristics using fluorescence intensity over time has been described (Yu et al.).

Another group of original research papers focused on how NGS approaches can be adapted to the diagnosis of infectious diseases and can empower clinicians to create personalized treatment strategies by providing comprehensive information about the infecting pathogen and its resistance profile in a timely manner. An original research paper showed how metagenomic NGS could represent a valuable supplement of conventional microbiological tests for the pathogen detection responsible of community acquired pulmonary infection (Lin et al.). A similar experience underscored that, in some cases metagenomic NGS (using Illumina short reads) was more sensitive than the conventional culture method in the detection of fastidious pathogens such as *Pneumocystis jirovecii* and Mucoraceae form blood samples, bronchoalveolar lavage fluid, cerebrospinal fluid, sputum, and ascitic fluid of immunocompromised patients (Li et al.). In another study the Nanopore sequencing approach (long reads) together with a set of multiple independent predictors, enabled the discrimination of uropathogens from colonizing bacteria in a large set of urinary tract infections (Jiang et al.). An original research provided insights on how metagenomic NGS can provide additional information to improve the diagnosis of *Cutibacterium acnes* orthopedic implant-associated infections. Taken together, metagenomic NGS was able to detect *C. acnes* DNA in more samples compared to culture and could be used to identify cases of suspected *C. acnes* orthopedic implant-associated infections, in particular, regarding possible polymicrobial infections, where the growth of *C. acnes* might be compromised due to a fast-growing bacterial species (Ponraj et al.).

Finally, a “METHODS article” published in this Research Topic showed how automated systems can empower healthcare facilities to establish robust surveillance programs for infectious diseases. In this paper a combination of real-time PCR assay with a strain typing method based on mid-infrared radiation associated with Fourier transform IR spectroscopy was adopted for rapid detection and typing of the emerging pathogen *Candida auris* (Contreras and Morgan).

As we move forward in the field of clinical microbiology, continuous research and development are vital. Challenges such as the integration of new technologies (metagenomic NGS), efficient data management, use of artificial intelligence and standardization of protocols need to be addressed (Hilt EE and Ferrieri, 2022; Egli, 2023). Moreover, ensuring accessibility to these advanced diagnostic tools in resource-limited settings is crucial.

The clinical impact of fast platforms and laboratory automation for the rapid diagnosis of infectious diseases, detection of antimicrobial resistance determinants, and diagnostic stewardship is profound. These technologies have transformed clinical microbiology, enabling healthcare providers to make quicker, more precise diagnoses and treatment decisions while aligning with the principles of diagnostic stewardship. As we continue to advance in this field, the potential to improve patient outcomes, reduce the spread of infectious diseases, combat antimicrobial resistance, and promote responsible diagnostic stewardship holds great promise, ushering in a brighter future for global healthcare.

## Author contributions

FA: Writing – original draft, Writing – review & editing. PB: Writing – review & editing. AM: Writing – review & editing.
